# Optimal Deployment of Charging Stations for Aerial Surveillance by UAVs with the Assistance of Public Transportation Vehicles

**DOI:** 10.3390/s21165320

**Published:** 2021-08-06

**Authors:** Hailong Huang, Andrey V. Savkin

**Affiliations:** 1 Department of Aeronautical and Aviation Engineering, Hong Kong Polytechnic University, Hong Kong 999077, China; hailong.huang@polyu.edu.hk; 2 School of Electrical Engineering and Telecommunications, The University of New South Wales, Sydney 2052, Australia

**Keywords:** drones, unmanned aerial vehicle (UAV), surveillance and monitoring, charging stations, public transportation vehicles, advances in robotic applications, robot sensing, vision-based sensing

## Abstract

To overcome the limitation in flight time and enable unmanned aerial vehicles (UAVs) to survey remote sites of interest, this paper investigates an approach involving the collaboration with public transportation vehicles (PTVs) and the deployment of charging stations. In particular, the focus of this paper is on the deployment of charging stations. In this approach, a UAV first travels with some PTVs, and then flies through some charging stations to reach remote sites. While the travel time with PTVs can be estimated by the Monte Carlo method to accommodate various uncertainties, we propose a new coverage model to compute the travel time taken for UAVs to reach the sites. With this model, we formulate the optimal deployment problem with the goal of minimising the average travel time of UAVs from the depot to the sites, which can be regarded as a reflection of the quality of surveillance (QoS) (the shorter the better). We then propose an iterative algorithm to place the charging stations. We show that this algorithm ensures that any movement of a charging station leads to a decrease in the average travel time of UAVs. To demonstrate the effectiveness of the proposed method, we make a comparison with a baseline method. The results show that the proposed model can more accurately estimate the travel time than the most commonly used model, and the proposed algorithm can relocate the charging stations to achieve a lower flight distance than the baseline method.

## 1. Introduction

Among the various promising applications of unmanned aerial vehicles (UAVs), aerial surveillance is the one that has attracted the greatest attention in recent years; it can be used for the protection of assets, people or objects, the investigation of crimes, and intelligence gathering. Aerial surveillance systems based on UAVs have become increasingly mature in these application tasks. They are not only cost-efficient, but also demonstrate high reliability and security [1]. This kind of multi-robot system can quickly explore large areas, such as mountainous regions, that are hard to reach for humans. It can significantly reduce the labour cost [2]. Thus, they have become an advanced means with which to replace human beings, efficiently and safely, in repeated surveillance missions [3]. Another main advantage of using UAVs for surveillance is the high probability of having a line-of-sight (LoS) with ground objects, which may be difficult to achieve with ground-based sensing units [4]. Extensive research has been conducted on the topics relevant for aerial surveillance, such as UAV video/image processing [5,6,7] and the deployment of UAVs [8,9,10,11]. The video/image processing techniques provide the visual sensing information required in various applications, and the approaches to the deployment of UAVs enable UAVs to find optimal/sub-optimal positions from which to conduct surveillance.

Although UAVs can achieve a high surveillance performance compared to other existing means, a challenging issue is the constrained flight time. In general, most of the commercial UAV products are powered by an onboard battery. Since the payload of a UAV is limited, the onboard battery cannot be very large, which results in a limitation of the capacity. Without carrying any other payload, a typical DJI Matrice 600 Pro model can only fly for 40 minutes with a fresh battery [12]. While improving the battery capacity and efficiency is indeed a solution, some other solutions have been investigated to increase the flight duration of UAVs. One promising solution is to install solar panels, which would enable UAVs to harvest solar energy [13] for flight time improvement. However, it is difficult to apply in complex environments, such as urban environments, due to the presence of many buildings. These can create shadows, and when UAVs fly into the shadows, their energy-harvesting rate reduces significantly. Another approach considers the usage of mobile chargers [14,15,16]. This method provides UAVs with the opportunity to replenish their battery, and it is more reliable than the solar-power harvesting approach. These mobile chargers, which are usually carried by some ground vehicles, are also controllable. However, in urban environments, these vehicles may create other issues—for example, finding where to park. Moreover, these vehicles may further worsen congested roads, if widely used. To alleviate the drawback of using mobile chargers, a method exploiting public transportation vehicles (PTVs) such as buses, trams and trains has been proposed [17,18,19,20]. Similar to human transportation within a city, a UAV can also travel with these vehicles as a passenger (on the roof of the vehicles). Differently to the mobile-charger approach, PTVs are existing vehicles travelling within urban areas. As such, this approach does not introduce extra vehicles to congested roads. When travelling with these vehicles, a UAV can simply turn off the motors to save energy. Thus, this approach has the potential to significantly improve the effective flight distance of UAVs.

**Figure 1:**
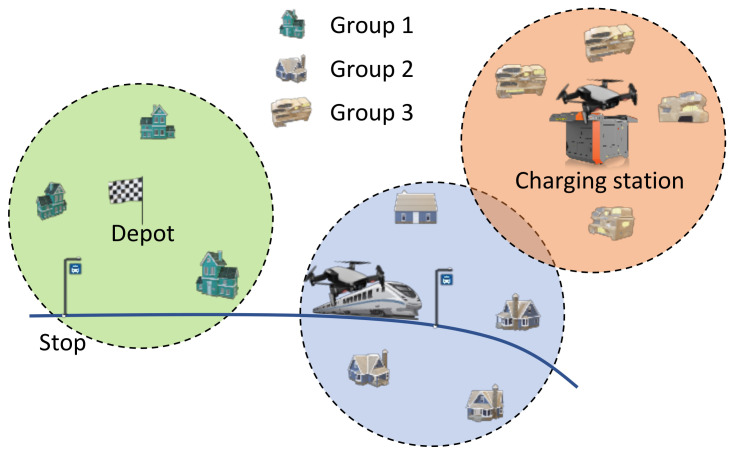
Illustration of the surveillance system and the grouping of sites based on their locations. The sites in the first group are located within a disk (in the ideal case) centred at the depot (green area). The sites in the second group are located within a disk centred at a vehicle stop (blue area). The sites in the third group are which are not covered by the first and second groups (orange area). A site located in the overlapping part of group two and group three can be reached by a UAV from a PTV.

We consider the example of using UAVs to survey several sites in a large-scale area. Practical applications include information broadcasting when wireless communication infrastructures are down, security/policing, traffic management, and parcel delivery [21,22]. We can classify the sites into three groups, based on their locations. The first group consists of sites that are within the flight range of UAVs departing from their depot. Any site in this group can be surveyed by a UAV without any other support. Roughly speaking, the sites in this group are located within a disk centred at the depot (see Figure 1); we call these sites the close sites. The second group consists of the sites that cannot be reached directly by UAVs from their depot, but can be reached with the support of PTVs. These sites are close to the routes of PTVs. If we assume that the UAVs can only leave and return to PTVs when the latter stops, and that the energy consumption when travelling with PTVs is negligible, the sites in the second group are roughly within the disks centred at these stops (see Figure 1). The sites in the second group are called relatively far sites. The sites in the third group are those cannot be reached, even with the support of PTVs. In other words, these sites are outside of the disks centred at the depot and the stops; see Figure 1. These are called far sites.

In this paper, we pay special attention to the surveillance of far sites, and we propose an approach that exploits PTVs and charging stations. In this approach, it is required to compute the travel time of UAVs from the depot to the sites. The travel time can be divided into two parts: one is the time spent travelling with PTVs, and the other is time spent travelling, with utilisation of the deployed charging stations, to reach the sites themselves. Considering that the travel time of PTVs is uncertain (due to various road traffic conditions), we can use the Monte Carlo method [23] to estimate the expected travel time with PTVs. Special attention is then paid to the deployment of charging stations. We propose a new coverage model to compute the travel time when UAVs travel through the charging stations to reach the sites. With this model, we formulate the optimal deployment problem with the goal of minimising the average travel time of UAVs from the depot to the sites, which can be regarded as a reflection of the quality of surveillance (QoS) (the shorter the better). We then propose a sub-optimal algorithm that relocates the charging stations iteratively. We show that this algorithm ensures that any movement of a charging station leads to a decrease in the average travel time of UAVs. To demonstrate the effectiveness of the proposed method, we make a comparison with a baseline method. The results show that the proposed model can more accurately estimate the travel time than the commonly used model, and the proposed algorithm can relocate the charging stations to achieve a lower flight distance than the baseline method.

The main contributions of this paper are summarised as follows:

- A new coverage model that can compute the travel time of UAVs from the depot to the sites more accurately than the commonly used model.
- A sub-optimal deployment method that guarantees that any relocation of a charging station leads to a decrease in the average travel time of UAVs.

The rest of the paper is organised as follows. Section 2 discusses the relevant publications, and we sketch where this paper stands in the literature. Section 3 introduces the system model and formulates the studied problem. Section 4 presents the proposed charging station deployment methods. Section 5 presents simulation results to demonstrate the effectiveness of the proposed methods. Finally, Section 6 concludes the paper and presents future research directions.

## 2. Related Work

In this section, we review the relevant publications and clarify the difference between the current paper and existing work.

There is a rich literature on UAV-based aerial surveillance on various types of targets, ranging from stationary areas to moving objects such as vehicles and humans [4,8,9,11]. For stationary areas, interesting theoretical research results have been presented in [8] for the deployment of UAVs to monitor objects in a given bounded area. An asymptotically optimal deployment, based on construction, is presented, which guarantees a minimum number of UAVs required to fully monitor the area of interest. For moving targets, with local knowledge but no global information, only locally optimal coverage of the moving targets can be achieved [11].

The problem considered in this paper relates to charging facility deployment for electric vehicles [24] and patrol station deployment for conventional vehicles. In general, the deployment of such facilities takes into account geographical conditions, because the facilities are relatively large. Alternatively, geographical conditions may not be necessary in the deployment of charging stations for UAVs—since such a charging station is small [25], it can even be deployed by roads. Charging facilities for electric vehicles are usually deployed in redundancy. That is, a charged electric vehicle can travel a distance that is much longer than the average distance between two charging facilities. Such redundancy is generally due to competition between different suppliers. In contrast, we deploy the charging stations for UAVs in a way that does not lead to unnecessary redundancy.

A UAV can either recharge or replace its battery at a charging station. It can then fly further, either to continue its current mission or to conduct other missions. Targeting the parcel-delivery application, the authors of [26] focus on a UAV station deployment problem. They consider a scenario where the customers are located far from a warehouse. A single UAV station is equipped with a fleet of UAVs, and it can be activated by the arrival of a truck that has parcels to be delivered. Then, the UAVs deliver the parcels to customers. The reference [27] formulates a charging station deployment optimisation problem with the goal of maximising the coverage of customers in a given area following the mixed-integer programming framework. A heuristic algorithm is presented for the setting where the feasible positions of charging stations are given by a discretised set. The work in [28] considers a similar problem, but the objective is to minimise the total system cost, including the charging stations, UAV ownership, service congestion, etc. In a different approach to [27,28], the paper [29] considers the deployment of charging stations in a continuous space, and a decentralised method is presented which can achieve the locally optimal coverage of customers. Targeting the application of inspecting a given set of sites, the reference [30] considers deploying charging stations at some sites so that a UAV can complete all the inspection tasks using the smallest amount of time; the authors additionally pay attention to the route planning problem for the UAV.

The problem that we will consider relates to the coverage control problem. Coverage control is a type of system partitioning and is an interesting problem in coordinated networks of mobile robots for environmental monitoring. A network of mobile robots is required to cover a region so that the event detection rate is maximised or the detection time is minimised [31]. The region of interest is often partitioned into a number of sub-regions (which is equal to the number of robots), and the robots can be optimally deployed in each sub-region [32]. Researchers have also investigated the coverage control for a group of heterogeneous robots [33]. The deployment of charging stations for UAVs shares the spirit of coverage control, in that all the sites in the area of interest should be reachable from at least one charging station. However, in the charging station deployment problem, the objective is to minimise the average time to reach the sites from the depot. Thus, the charging station deployment problem has a tree-like structure, while the general coverage control problem can be fully distributed.

In a different, yet related context, i.e., UAV video/image processing, existing publications have studied processing the videos taken by UAVs when they are at some fixed position. From simple to complex, several typical problems have been investigated, such as road detection [34], vehicle detection and tracking [35], and traffic parameter extraction [36]. Popular methods include the use of convolutional neural networks (CNNs) [37] to analyse individual frames, as well two consecutive frames, of a UAV’s footage [38]. However, these approaches are only suitable for the case where the UAV can hover at a certain attitude. Indeed, this state is often difficult to achieve, especially when there is a wind gust. To address this, some researchers have investigated the ego-motion issue as generated either intentionally, by a remote operator, or as an external factor, such as caused by the wind. The first approach to this problem is called the image registration [39]. This is a method that attempts to turn the moving background into a fixed background, which allows traditional methods for background subtraction to be used. The second approach is based on optical flow [40], which extracts the motion pattern from videos and is often combined with unsupervised learning for estimating the UAV ego-motion. Thus, the video/image processing approaches provide fundamental application-dependent sensing information.

## 3. Problem Statement

We consider an aerial surveillance system with several key components: a depot, a fleet of UAVs, a set of sites, PTVs, and charging stations. The UAVs are energy-constrained. We assume that a PTV can accept one UAV on its roof. The sites to be surveyed are distributed in an urban area, and they are classified into three groups as discussed in Section 1. We only focus on the surveillance of the sites in the third group. Table A1 summarises the main symbols used in the paper.

The problem under investigation is the optimal deployment of charging stations, so that the sites of interest can be surveyed by UAVs in the shortest time (a reflection of the QoS). In particular, we consider a remote area that contains a vehicle stop. Departing from the depot (denoted by *D*), a UAV can reach this stop by boarding some number of PTVs. We focus on deploying a number *n* of charging stations in this remote area. For simplicity, an additional charging station (denoted by $p_{0}$) is deployed at the vehicle stop. Suppose that a fully charged battery allows a UAV to fly for a distance of 2R. The charging station at the vehicle stop serves the sites in a circular area centred at $p_{0}$ of radius *R*—see the blue area in Figure 1. Then, our focus is on finding locations for the other *n* charging stations. Let $p_{1},\ldots ,p_{n}$ denote the locations of these *n* charging stations, which are to be decided. It is worth pointing out that this paper focuses on the high-level planning problem. The relevant low-level control issues, such as the UAV dynamics, the accuracy of UAV positioning and landing, and external disturbances (gust and wind) are not considered in the paper.

The public transportation network, the depot, the deployed charging stations, and the sites form a graph, which can be used to plan paths for UAVs to survey the sites. Let G(V,E) denote this graph. The vertex set *V* consists of the depot *D*, the vehicle stops, charging stations $p_{1},\ldots ,p_{n}$, and the sites. The edge set *E* contains directed links from one stop node to another, representing public transportation services between them. It also contain undirected links between any two stop nodes, any two charging station nodes, and any pair of a charging station node and a site node—these represent UAV flight between these locations. It is worth mentioning that if two charging station nodes $p_{i}$ and $p_{j}$ (i≠j,i,j∈[0,n]) are connected in the graph *G*, we have

(1) $$|p_{i},p_{j}|\leq 2R,$$

where |·,·| gives the standard Euclidean distance between two points. It is also worth mentioning that any site node is connected to a charging station node within range *R*. Note that the flight range *R* corresponds to flight along straight lines. If a UAV encounters some obstacle, such as a building, this straight line assumption is not valid. However, we can avoid this issue by setting the flight altitude to be higher than any local buildings.

**Figure 2:**
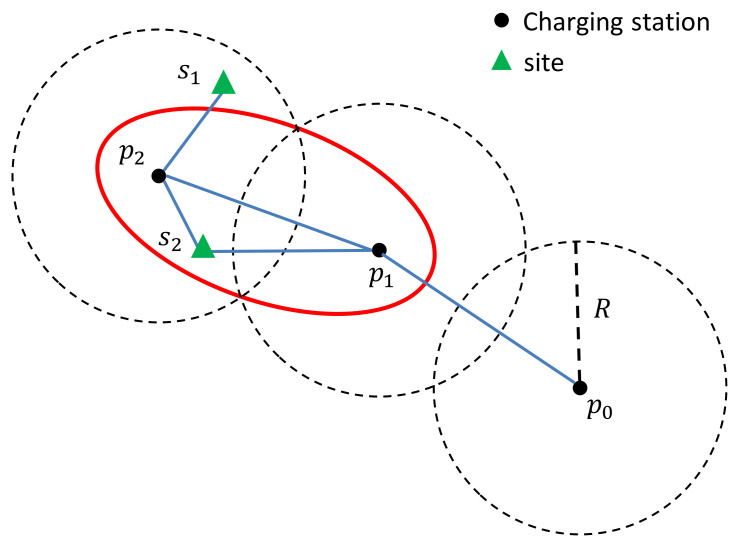
Illustrative example of the connections between site nodes and charging station nodes. Although both $s_{1}$ and $s_{2}$ are located within the circle centred at $p_{2}$, $s_{1}$ connects with $p_{2}$ but not $p_{1}$, while $s_{2}$ connects with both $p_{1}$ and $p_{2}$. This is because $|p_{1},s_{1}\left|+\right|s_{1},p_{2}|>2R$, while $|p_{1},s_{2}\left|+\right|s_{2},p_{2}|\leq 2R$.

Moreover, a site node can be connected with another charging station node if the sum of distances to the two charging station nodes is no larger than 2R. In the example shown in Figure 2, the top site $s_{1}$ only connects with the charging station node $p_{2}$, while the bottom site $s_{2}$ connects with the charging station nodes $p_{1}$ and $p_{2}$. We note that if a site connects with two charging stations, it is located inside an ellipse with these charging stations as the foci and passes the intersection points of the circles centred at these charging stations of radius *R*; see Figure 2. In the graph *G*, a site is reachable from any charging station connecting with the site. In Figure 2, $s_{1}$ can be reached from the charging station $p_{2}$, while $s_{2}$ can be reached from the charging stations $p_{1}$ and $p_{2}$. Constructing the graph *G* in this way leads to a shorter delivery time for sites such as $s_{2}$ in Figure 2. If $s_{2}$ is not connected with $p_{1}$, but only with $p_{2}$, in the graph *G*, a UAV has to fly to $p_{2}$ and then to $s_{2}$. Certainly,

(2) $$|p_{1},p_{2}\left|+\right|p_{2},s_{2}\left|\geq \right|p_{1},s_{2}|.$$

Note that connecting $s_{2}$ with $p_{1}$ does not mean that we can disconnect $s_{2}$ and $p_{2}$. The link between $s_{2}$ and $p_{2}$ is necessary, as it enables the UAV to return after completing the delivery. This is because,

(3) $$|p_{1},s|+|s,p_{2}|\leq 2R.$$

Let *S* denote the set of sites in the area of interest. Let $\rho_{s}\left(t\right)$ denote the probability that a request to survey site s∈S is placed at time *t* of a day. This probability is highly application-dependent and can be easily obtained from historical data. Let $t_{s}$ and $t_{f}$ be the earliest time and the latest time of placing a survey request, respectively. Then, we have $\int_{t_{s}}^{t_{f}}\rho_{s}\left(t\right)dt=1$. In addition, let $\lambda_{s}$ be the weight of the site s∈S. This weight is the frequency of surveying the site. For two sites, the weight of the more important site is larger, and as such it needs to be surveyed more frequently than the other. We assume that both the probability and the weight of a site are known.

We now introduce a function, τ(u,v,t), which computes the shortest travel time from node u∈V to node v∈V when a UAV departs from node *u* at time *t*. If the nodes *u* and *v* are not connected, neither directly nor via any other nodes in *G*, τ(u,v,t)=∞,∀t. In practice, the travel time of an edge in the graph *G* can be different at different times. Even at the same time on different days, the duration of travel can be different due to the stochastic nature of public transportation networks. Since the travel time of the shortest path between two nodes may vary over time and be uncertain, so too for the graph *G*. The time-variance of *G* can be reflected by the timetables of public transportation services. The uncertainty of these services, due to factors such as congestion, can be modelled as random noise. We follow the label-setting algorithm used in [20] to construct the shortest path between two nodes, and then adopt Monte Carlo simulations [23] to account for the uncertainty. The Monte Carlo method is an ideal method for computing probabilities and expectations when the analytical integration is impossible or impractical. Although the Monte Carlo estimation is not exact, we understand that when a sufficiently large number of random variables are generated, the error of approximation can become arbitrarily small. In the considered context, by generating a sufficient number of simulations, we can obtain the mean travel time between nodes *u* and *v* for a particular starting time *t*. Without introducing a new symbol, we use τ(u,v,t) in the rest of the paper to represent this mean travel time. We consider that a UAV replaces its battery at a charging station, and that the charging stations organise the charging of the used battery during spare time. Since the time for such a replacing operation can be much shorter than the flight time between two charging stations or the travel time between two vehicle stops, it is assumed to be negligible.

With this function, we can represent the average time required to survey a site when departing from the depot. Specifically, the average time for a UAV to reach a site *s*, when the UAV leaves the depot at time *t*, can be represented by τ(D,s,t). As the starting time, i.e., the time a site surveillance request is activated, may be uncertain, we consider the mathematical expectation of the average travel time for surveying site *s*, which is denoted by $T_{s}\left(p_{1},\ldots ,p_{n}\right)$ and is given as follows:

(4) $$T_{s}\left(p_{1},\ldots ,p_{n}\right)=\int_{t_{s}}^{t_{f}}\rho_{s}\left(t\right)\tau \left(D,s,t\right)dt.$$

Here, $\left[t_{s},t_{f}\right]$ is the time window during which a site surveillance request can be activated. Note that the average travel time for a site *s*, i.e., $T_{s}\left(p_{1},\ldots ,p_{n}\right)$, is now represented by a function of the locations of charging stations, i.e., $p_{1},\ldots ,p_{n}$. It is also worth noting that such an average travel time also depends on the edges in *G*, which represent the public transportation services. The reason for not including this factor as an adjustable variable is that the public transportation services are not controllable by a supplier. Instead, from the point of a supplier, only the locations of the charging stations, i.e., $p_{1},\ldots ,p_{n}$, can be controlled.

**Problem Statement:** The charging station deployment problem aims to find the locations of the charging stations, i.e., $p_{1},\ldots ,p_{n}$, that minimise the weighted average travel time of the sites. It can be formulated as follows:

(5) $$\min_{p_{1},\ldots ,p_{n}}\sum_{s\in S}\lambda_{s}T_{s}\left(p_{1},\ldots ,p_{n}\right).$$

There are some constraints that the deployment needs to satisfy. Firstly, any site *s* must be covered by at least one charging station, i.e.,

(6) $$|s,p_{i}|\leq R,\exists i\in \left[1,n\right],\forall s\in S.$$

Moreover, every charging station is connected with $p_{0}$, i.e.,

(7) $$\tau \left(p_{i},p_{0},0\right)<\infty ,\forall i\in \left[1,n\right].$$

Note that in (7) we can set the time, i.e., the third entry of the function τ(u,v,t), as any value—not only zero.

**Remark** **1.** *Note that for a site, the charging station that covers it is not necessarily on the shortest path for a UAV to serve it. For example, in Figure 2, a UAV serves the site $s_{2}$ via $p_{1}$, not $p_{2}$. Connecting $s_{2}$ with $p_{2}$ enables the UAV to return after completing the delivery, because $|p_{1},s|+|s,p_{2}|\leq 2R$. Moreover, constructing the graph G in the aforementioned way leads to a shorter travel time for sites such as $s_{2}$ in Figure 2. We can imagine that if $s_{2}$ is not connected with $p_{1}$, but only with $p_{2}$, in the graph G, a UAV has to fly to $p_{2}$ and then to $s_{2}$. This would lead to a longer travel time; see (2). This issue requires us to clarify the charging station via which each site is surveyed by a UAV in the shortest time.*

## 4. Proposed Method

In this section, we discuss the proposed method to address the problem presented by (5)–(7). We start with the introduction of two basic concepts used throughout the development of our approach (Section 4.1). Then, we propose the deployment method for the simple case with a single charging station (Section 4.2), then for the complex case with multiple charging stations (Section 4.3).

### 4.1. Coverage Models

We introduce the following two fundamental concepts.

**Definition** **1** (The charging stations that cover a site)**.** *A site s is said to be covered by a charging station $p_{i}$ if one of the below two conditions holds:*

- *The distance between the site s and the charging station $p_{i}$ is no more than R, i.e., $|p_{i},s|\leq R$ [41].*
- *There exists another charging station $p_{j}$ such that the summation of the distances between the two charging stations and the site is no greater than 2R, i.e., $|p_{i},s|+|p_{j},s|\leq 2R$.*

**Definition** **2** (The charging station to survey a site)**.** *A site s is said to be surveyed from a charging station $p_{i}$ if the charging station $p_{i}$ is the last charging station on the shortest path from the depot to the site s in the graph G.*

From the above two definitions, we can see that there may exist multiple charging stations that cover a site. However, generally, there is only one charging station surveying a site, since the charging station that surveys a site should be the last one on the shortest path–see Definition 2. We also understand that the charging station via which a site is surveyed also covers the site. However, the charging station that covers a site is not necessarily the one via which to survey the site. Typical examples are given in Figure 2. The site $s_{1}$ is covered by the charging station $p_{2}$, and the site $s_{2}$ is covered by the charging stations $p_{1}$ and $p_{2}$. The site $s_{1}$ is surveyed via the charging station $p_{2}$ (the only charging station that covers $s_{1}$), while the site $s_{2}$ is surveyed via the charging station $p_{1}$ (one of the charging stations that cover $s_{2}$).

Definition 1 reveals an interesting and useful property. That is, if a site is located within an ellipse formed by two charging stations (with the positions of the two charging stations as the foci, and with the ellipse passing the intersection points of the two *R*-radius circles that centred at the charging stations), this site can be covered by more than one charging station. Again, taking Figure 2 as an example, site $s_{2}$ is inside such an ellipse (see the red one around $p_{1}$ and $p_{2}$), while site $s_{1}$ is not. Thus, site $s_{2}$ is covered by two charging stations.

It should be pointed out that Definition 1 is superior to other existing definitions, which are based on only the first condition of Definition 1 (see [41] and the references therein), with respect to the travel time of a UAV from the depot to a site. This is crucial when the site is located within one of the aforementioned ellipses, i.e., being covered by more than one charging station, because the second condition promises to result in a shorter path through which a UAV can conduct the surveillance mission. In particular, if only the first condition of Definition 1 is considered, the site $s_{2}$ in Figure 2 is covered by the charging station $p_{2}$. Then, when we plan the path for a UAV to survey the site $s_{1}$, we will find the path $D\to \cdots \to p_{0}\to p_{1}\to p_{2}\to s_{2}$. However, with the second condition of Definition 1, the site $s_{2}$ is also connected with $p_{1}$ in the graph *G*. Thus, the path for a UAV can be $D\to \cdots \to p_{0}\to p_{1}\to s_{2}$. Clearly, $|p_{1},p_{2}\left|+\right|p_{2},s_{2}\left|\geq \right|p_{1},s_{2}|$, where the equality holds only when $s_{2}$ is located on the line segment connecting $p_{1}$ and $p_{2}$. With Definition 2, no matter how many nodes are connected to a site *s* in the graph *G*, we can deduce the charging station via which to survey the site.

In the subsequent sections, we will use these definitions to develop our approach for deploying charging stations.

### 4.2. Deployment of a Single Charging Station

In this section, we present the method for deploying a single charging station.

As mentioned in Section 3, $p_{0}$ has already been deployed at a vehicle stop near the area of interest. Thus, we consider the deployment of the second charging station $p_{1}$. One necessary assumption in this simple scenario is that the sites to be surveyed are located somewhere such that there exist feasible positions for the second charging station to serve the sites. Otherwise, there is no solution to the deployment problem. A geometric view of this assumption is that the sites are close to a vehicle stop, and they are not distributed very sparsely. Moreover, there are some sites that are outside the disk with radius of *R* centred at $p_{0}$. Otherwise, the charging station $p_{0}$ is sufficient to serve them.

**Figure 3:**
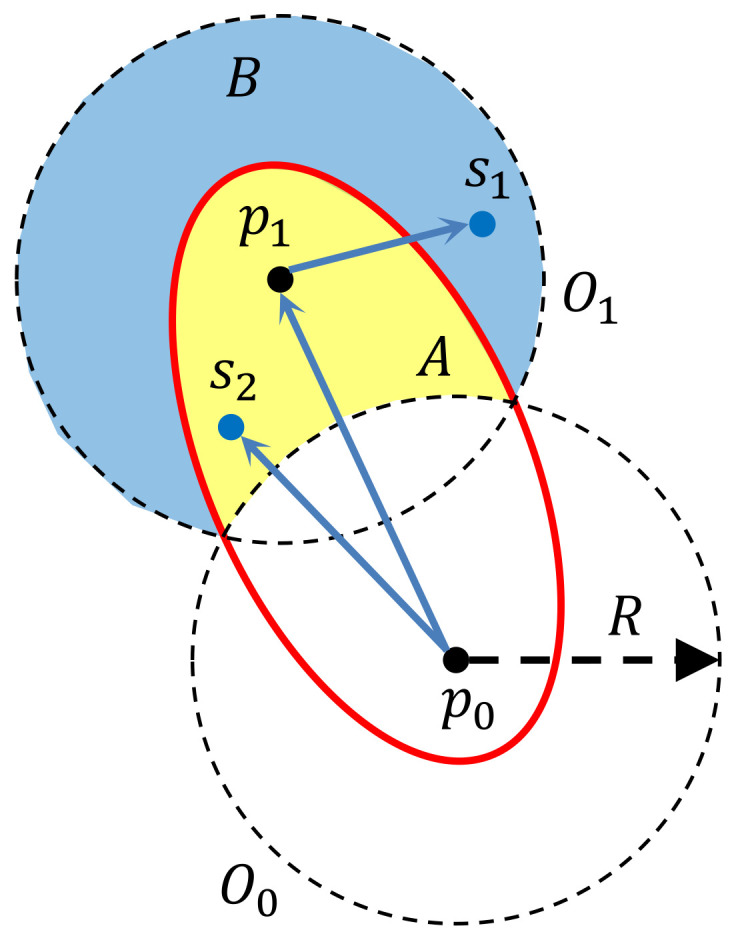
Illustration of the coverage of two charging stations $p_{0}$ and $p_{1}$.

Given the positions of $p_{0}$ and $p_{1}$, we first construct the circles centred at $p_{0}$ and $p_{1}$ with the radius of *R*, which are denoted by $O_{0}$ and $O_{1}$, respectively. As discussed in Section 4.1, we can construct an ellipse with $p_{0}$ and $p_{1}$ as the foci which passes through the intersections of the circles $O_{0}$ and $O_{1}$—see Figure 3. Such an ellipse divides the disk centred at $p_{1}$ into two parts. The first one is the part inside the ellipse, denoted by *A*—see the yellow part in Figure 3. The second one is the part outside the ellipse, denoted by *B*—see the blue part in Figure 3. By Definition 1, the sites in *A* are covered by both $p_{0}$ and $p_{1}$, while those in *B* are only covered by $p_{1}$. For the sites in *A*, according to Definition 2, it is $p_{0}$ that serves them, while for those in *B*, it is $p_{1}$ that serves them.

For a site s∈A, the average travel time can be computed by:

(8) $$\begin{array}{cc} T_{s}\left(p_{1}\right) & =\int_{t_{s}}^{t_{f}}\tau \left(D,p_{0},t\right)dt+\int_{t_{s}}^{t_{f}}\rho_{s}\left(t\right)\left|p_{0},s\right|dt\\ \; & =\int_{t_{s}}^{t_{f}}\tau \left(D,p_{0},t\right)dt+\left|p_{0},s\right|. \end{array}$$

In (8), $T_{s}\left(p_{1}\right)$ is the average flight time from the depot *D* to site s∈A. We only take $p_{1}$ as an input, since we consider the deployment of this single charging station. $\tau (D,p_{0},t)$ is the travel time from the depot *D* to the charging station $p_{0}$ when the UAV departs at time *t*. Then, $\int_{t_{s}}^{t_{f}}\tau \left(D,p_{0},t\right)dt$ gives the mathematical expectation of this travel time. Moreover, $\int_{t_{s}}^{t_{f}}\rho_{s}\left(t\right)|p_{0},s|dt=|p_{0},s|$, because $|p_{0},s|$ is independent of time *t* and $\int_{t_{s}}^{t_{f}}\rho_{s}\left(t\right)dt=1$.

Similarly, for a site s∈B, the average travel time can be computed by:

(9) $$\begin{array}{cc} T_{s}\left(p_{1}\right) & =\int_{t_{s}}^{t_{f}}\tau \left(D,p_{0},t\right)dt+\int_{t_{s}}^{t_{f}}\rho_{s}\left(t\right)\left(\right|p_{0},p_{1}\left|+\right|p_{1},s\left|\right)dt\\ \; & =\int_{t_{s}}^{t_{f}}\tau \left(D,p_{0},t\right)dt+|p_{0},p_{1}\left|+\right|p_{1},s|. \end{array}$$

Then, the weighted average travel time for all sites in *S* is given by:

(10) $$\begin{array}{cc} \; & \frac{1}{\left|S\right|}(\sum_{s\in A}\lambda_{s}(\int_{t_{s}}^{t_{f}}\tau \left(D,p_{0},t\right)dt+|p_{0},s|)+\sum_{s\in B}\lambda_{s}(\int_{t_{s}}^{t_{f}}\tau \left(D,p_{0},t\right)dt+|p_{0},p_{1}\left|+\right|p_{1},s\left|\right))\\ = & \int_{t_{s}}^{t_{f}}\tau \left(D,p_{0},t\right)dt+\frac{1}{\left|S\right|}(\sum_{s\in A}\lambda_{s}|p_{0},s|+\sum_{s\in B}\lambda_{s}\left(\right|p_{0},p_{1}\left|+\right|p_{1},s\left|\right)) \end{array}$$

From (10) we can see that, given the first charging station $p_{0}$, the weighted average travel time for the sites in *S* only depends on the position of the second charging station $p_{1}$, since the mathematical expectation of the travel time from the depot *D* to $p_{0}$ is fixed when given $p_{0}$ and *G*.

Therefore, the problem of deploying one charging station is reformulated as:

(11) $$\min_{p_{1}}\frac{1}{\left|S\right|}(\sum_{s\in A}\lambda_{s}|p_{0},s|+\sum_{s\in B}\lambda_{s}\left(\right|p_{0},p_{1}\left|+\right|p_{1},s\left|\right))$$

subject to

(12) $$|s,p_{1}|\leq R,\forall s\in S.$$

It should be pointed out that we only need to consider the sites that are outside the circle $O_{0}$. The reason is that, given $p_{0}$, the travel time for the sites in $O_{0}$ is known, which does not need to be optimised.

The main difficulty in addressing the above problem lies in its discontinuity. The variable $p_{1}$ not only determines the flight time in part *B*, but also impacts the parts *A* and *B*. $p_{1}$ is defined in a continuous space. A small change of $p_{1}$ may lead to different parts *A* and *B*, which may result in a large jump in terms of the travel time. Consider a site that is on the boundary of *A* and *B*. In this case, the site belongs to part *A*. However, when we move $p_{1}$ a small amount, it is possible that this site falls into part *B*. Clearly, this creates a jump in the travel time of this site, which further leads to a discontinuity in the overall weighted travel time.

To address the problem, we reformulate it as an integer linear problem (ILP). Suppose there is a set of candidate positions where we are allowed to deploy the charging station. Let $C=\{c_{1},c_{2},\ldots ,c_{m}\}$ denote such a set. Let $x_{k}$ be a binary variable. $x_{k}=1$ if the charging station is located at the candidate $c_{k}$; $x_{k}=0$ otherwise. Moreover, given these candidates, we can constructs the parts *A* and *B* correspondingly. For the candidate $c_{k}$, let $A_{k}$ and $B_{k}$ denote the corresponding parts. Then, the problem is formulated as follows:

(13) $$\min_{x_{1},\ldots ,x_{m}}\sum_{k=1}^{m}x_{k}(\sum_{s\in A_{k}}\lambda_{s}|p_{0},s|+\sum_{s\in B_{k}}\lambda_{s}\left(\right|p_{0},p_{1}\left|+\right|p_{1},s\left|\right))$$

subject to

(14) $$\sum_{k=1}^{m}x_{k}=1,$$

(15) $$x_{k}\in \left\{0,1\right\},\forall k=1,\ldots ,m.$$

It is worth pointing out that the above formulation constraint (12) has already been taken into account in the process of constructing the parts $A_{k}$ and $B_{k}$. If a candidate position cannot enclose all the sites to be surveyed, it has already been removed from the candidate list.

Now, the problem is in the well-known form of an ILP, and many existing solvers are available for this purpose.

### 4.3. The Deployment of Multiple Charging Stations

In this section, we focus on the more challenging scenario with *n* charging stations, i.e., $p_{1},\ldots ,p_{n}$.

Suppose the charging stations are initially deployed at $p_{1},\ldots ,p_{n}$, and the two aforementioned conditions hold, i.e., all the sites are covered by at least one charging station (see (6)), and each charging station is connected with $p_{0}$ (see (7)). With these assumptions, we can construct a tree structure for the charging stations, including $p_{0}$. In particular, $p_{0}$ is the root of this tree, and any other vertex connects with $p_{0}$ via the shortest path. This is the so-called minimum spanning tree. For any vertex *j* in the tree, let $P_{j}$ denote the shortest path from the root $p_{0}$ to vertex *j*. Clearly, this path gives the shortest travel time for UAVs. Moreover, let $|P_{j}|$ denote the number of vertices on this path, and let $P_{j}\left[k\right]$ denote the *k*th vertex on the path. In particular, $P_{j}\left[1\right]=p_{0}$, and $P_{j}\left[\right|P_{j}\left|\right]=p_{j}$. With this symbol, the requirement that any charging station must connect with $p_{0}$ can be formulated as:

(16) $$|P_{j}\left[k\right],P_{j}\left[k+1\right]|\leq 2R,\forall k=1,\ldots ,|P_{j}|-1.$$

Moreover, let ϕ(j) denote the parent vertex of vertex *j* on the path $P_{j}$. It is clear that in the minimum spanning tree, any vertex other than the root has a unique parent vertex, while each vertex may have more than one child vertex. Let ψ(j) denote the set of child vertices of vertex *j*.

**Figure 4:**
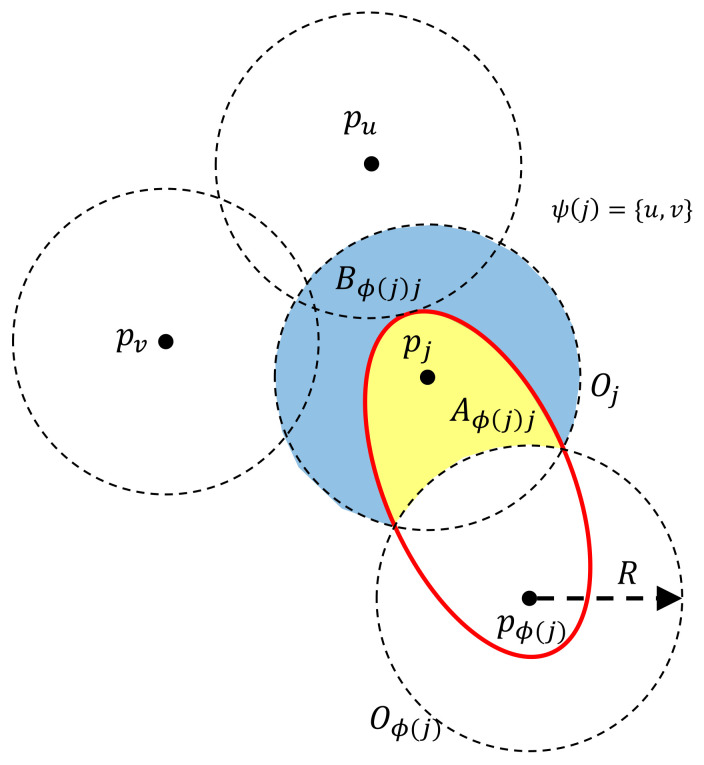
Illustration of the coverage of a pair of child-parent charging stations $p_{j}$ and $p_{\phi (j)}$. Vertex ϕ(j) is the parent of vertex *j*, and the set of the child vertices of Vertex *j* consists of *u* and *v*, i.e., ψ(j)={u,v}.

Now, for any pair of child-parent vertices, we can construct the corresponding ellipse following the method discussed in Section 4.2. It is worth pointing out that there always exists such an ellipse for a pair of child-parent vertices if their distance is less than 2R. For the extreme case, i.e., their distance equals 2R, such an ellipse reduces to a line segment connecting the child and parent charging stations. Similarly to Section 4.2, let $O_{j}$ and $O_{\phi (j)}$ denote the circles centred at $p_{j}$ and $p_{\phi (j)}$ of radius *R*, respectively. Let $A_{\phi (j)j}$ and $B_{\phi (j)j}$ denote the two parts of $O_{j}$ separated by the ellipse constructed from $p_{j}$ and $p_{\phi (j)}$, see Figure 4.

For the sites in the part of $A_{\phi (j)j}$, the travel time for UAVs is broken down into three legs:

1. from *D* to $p_{0}$,
2. from $p_{0}$ to $p_{\phi (j)}$,
3. from $p_{\phi (j)}$ to the sites.

For the sites in the part of $B_{\phi (j)j}$, the travel time for UAVs is broken down into four legs:

1. from *D* to $p_{0}$,
2. from $p_{0}$ to $p_{\phi (j)}$,
3. from $p_{\phi (j)}$ to $p_{j}$.
4. from $p_{j}$ to the sites.

Similarly to Section 4.2, we only pay attention to the travel time from $p_{0}$ to the sites. For ease of presentation, let $F_{1}\left(p_{0},p_{\phi (j)}\right)$ denote the travel time from $p_{0}$ to $p_{\phi (j)}$. Let $S_{j}$ denote the subset of sites within the circle $O_{j}$. Let $F_{2}\left(p_{\phi (j)},p_{j}\right)$ be the average travel time from $p_{\phi (j)}$ to the sites in $S_{j}$. Then, we have

(17) $$F_{2}\left(p_{\phi (j)},p_{j}\right)=\frac{1}{|S_{j}|}(\sum_{s\in A_{\phi (j)j}}|p_{\phi (j)},s|+\sum_{s\in B_{\phi (j)j}}\left(\right|p_{\phi (j)},p_{j}\left|+\right|p_{j},s\left|\right)).$$

The deployment of multiple charging stations can then be obtained by solving the below problem:

(18) $$\min_{p_{1},\ldots ,p_{n}}\frac{1}{n}\sum_{j=1}^{n}\left(F_{1}\left(p_{0},p_{\phi (j)}\right)+F_{2}\left(p_{\phi (j)},p_{j}\right)\right).$$

Clearly, (18) gives the average travel time of UAVs from $p_{0}$ to the sites.

The direct method to solve the above problem is to convert the problem to its discrete version, as used for the simple case with just one charging station. However, such a method may not greatly simplify the problem—in addition to the variables that decide which candidate locations are chosen for charging station deployment, we must also introduce variables to satisfy the connectivity requirement for each charging station. Clearly, this will make the corresponding ILP contain much more variables; as such, an existing ILP solver may take a very long time to finish analysing the problem. We therefore present a sub-optimal method that can solve the problem quickly.

We observe that the movement of a vertex in the constructed tree influences the travel time to the sites covered by itself, its child, and its downstream vertices. However, this movement does not impact that of the sites covered by upstream vertices. Thus, moving a vertex only affects the travel time of the sites covered by its branch; we will call the vertex that we consider the sub-root of the branch. Another observation is that the vertices in a branch can be classified into two groups: the neighbour vertices of the sub-root, that are directly connected with the sub-root (this group includes the sub-root itself); and the non-neighbour vertices, that are not connected with the sub-root directly. For non-neighbour vertices, the movement of the sub-root only affects the first part in (18), but not the second part. We can call this impact the indirect impact. For the neighbour vertices, the movement of the sub-root affects both parts in (18). In this case, the impact is named the direct impact. In the method to be presented, we relocate a vertex by taking into account both its direct and indirect impacts. In particular, a vertex is relocated to another position if the overall impact provides a decrease in the average travel time to the sites in its branch. We move the vertex to the position that can obtain the maximum decrease in the average travel time.

As per our discussion, the relocation of a leaf vertex (a vertex that does not have any child vertices ) can be achieved using the method discussed in Section 4.2 because a leaf vertex’s movement only impacts the sites covered by itself. However, the relocation of a non-leaf vertex is complex. To present the relocation method for the latter case, we need to introduce some more symbols. Let $N_{i}$ denote the number of the sites in the branch of vertex *i* which are indirectly impacted by vertex *i*. Let $G_{i}$ denote the average travel time from vertex *i* to these sites. If *i* is a leaf vertex or the parent of a leaf vertex, $N_{i}=0$ and $G_{i}=0$, because the sites in the branch of vertex *i* are all directly impacted by vertex *i*. Let $H_{1}\left(p_{j}\right)$ denote the average travel time from the parent of vertex *j*, i.e., ϕ(j), to all the indirectly impacted sites in the branch of vertex *j*. If $\sum_{i\in \psi (j)}N_{i}=0$, $H_{1}\left(p_{j}\right)=0$; otherwise, $H_{1}\left(p_{j}\right)$ is computed as follows:

(19) $$H_{1}\left(p_{j}\right)=\frac{\sum_{i\in \psi (j)}N_{i}(G_{i}+|p_{i},p_{j}\left|+\right|p_{j},p_{\phi (j)}\left|\right)}{\sum_{i\in \psi (j)}N_{i}}.$$

In (19), the term $G_{i}+|p_{i},p_{j}\left|+\right|p_{j},p_{\phi (j)}|$ gives the average travel time from vertex ϕ(j) to the sites in the branch of vertex *i* (where i∈ψ(j)) that are indirectly impacted by vertex *j*. Clearly, $H_{1}\left(p_{j}\right)$ is a function of $p_{j}$, and other information in (19), including $N_{i}$, $G_{i}$, and $p_{\phi (j)}$, is known if the downstream vertices and the parent vertex are fixed.

Let $H_{2}\left(p_{j}\right)$ be the average travel time from vertex ϕ(j) to the sites that are in the branch of node *j* and are directly impacted by vertex *j*. These sites include the ones covered by vertex *j* and the child vertices of vertex *j*. The number of the sites is $|S_{j}|+\sum_{i\in \psi (j)}\left|S_{i}\right|$. Then, $H_{2}\left(p_{j}\right)$ is computed as follows:

(20) $$\begin{array}{cc} H_{2}\left(p_{j}\right)= & \frac{1}{|S_{j}|+\sum_{i\in \psi (j)}\left|S_{i}\right|}[\sum_{s\in A_{\phi (j)j}}|p_{\phi (j)},s|+\sum_{s\in B_{\phi (j)j}}\left(\right|p_{\phi (j)},p_{j}\left|+\right|p_{j},s\left|\right)+\\ \; & \sum_{i\in \psi (j)}\left(\sum_{s\in A_{ji}}|p_{j},s|+\sum_{s\in B_{ji}}\left(\right|p_{j},p_{i}\left|+\right|p_{i},s|)+\sum_{s\in S_{i}}\left|p_{j},p_{\phi (j)}\right|\right)]. \end{array}$$

The term $\sum_{S\in A_{\phi (j)j}}|p_{\phi (j)},s|+\sum_{s\in B_{\phi (j)j}}\left(\right|p_{\phi (j)},p_{j}\left|+\right|p_{j},s\left|\right)$ in (20) gives the total travel time from vertex ϕ(j) to the sites in $S_{j}$. The term $\sum_{s\in A_{ji}}|p_{j},s|+\sum_{s\in B_{ji}}\left(\right|p_{j},p_{i}\left|+\right|p_{i},s|)+\sum_{s\in C_{i}}\left|p_{j},p_{\phi (j)}\right|$ in (20) gives the total travel time from vertex ϕ(j) to the sites in $S_{i}$, where i∈ψ(j). Clearly, $H_{2}\left(p_{j}\right)$ is a function of $p_{j}$, and other information in (20) is known if the downstream vertices and the parent vertex are fixed. Differently from $H_{1}\left(j\right)$, which can be zero, $H_{2}\left(j\right)>0$, because there exists a subset of sites that are directly impacted by vertex *j*. If this subset is empty, there is no need to have vertex *j*.

Let $H(p_{j})$ denote the average travel time from vertex ϕ(j) to all the indirectly and directly impacted sites covered by vertex *j*. Then,

(21) $$H\left(p_{j}\right)=\frac{\left(\right|S_{j}|+\sum_{i\in \psi (j)}|S_{i}\left|\right)H_{2}\left(p_{j}\right)+\sum_{i\in \psi (j)}N_{i}H_{1}\left(p_{j}\right)}{|S_{j}|+\sum_{i\in \psi (j)}\left|S_{i}\right|+\sum_{i\in \psi (j)}N_{i}}.$$

Moreover, the average travel time from vertex 0 to all the sites in the branch of vertex *j* is given by $L\left(p_{0},p_{\phi (j)}\right)+H\left(p_{j}\right)$, where $L(p_{0},p_{\phi (j)})$ denotes the travel time from vertex 0 to vertex ϕ(j). If at any time we only move one vertex in the constructed tree, say vertex *j*, for the purpose of reducing the average travel time, we only need to consider $H(p_{j})$, because $L(p_{0},p_{\phi (j)})$ is fixed. Therefore, we can relocate $p_{j}$ to a new place that minimises (21), provided that the topology of the constructed tree remains and vertex *j* does not lose any of its covered sites.

Moreover, since the values of *N* and *G* are required in the computation of $H_{1}\left(p_{j}\right)$ in (19), we need a general formula for them to relocate each node in a decentralised manner. Given the values of *N* and *G* of the child vertices of vertex *j*, we can compute these values for vertex *j* as follows:

(22) $$N_{j}=\sum_{i\in \psi (j)}(N_{i}+\sum_{k\in \psi (i)}|S_{k}\left|\right),$$

(23) $$\begin{array}{cc} G_{j}=\frac{1}{N_{j}} & \sum_{i\in \psi (j)}\left(N_{i}(G_{i}+|p_{i},p_{j}\left|\right)+\sum_{s\in A_{ji}}|p_{j},s|+\sum_{s\in B_{ji}}\left(\right|p_{j},p_{i}\left|+\right|p_{i},s\left|\right)\right). \end{array}$$

The values of *N* and *G* propagate in upstream order. For a certain vertex, these values can be computed once those of its child vertices have been computed.

Now, we have enough symbols to present our method. Suppose that at the initial positions $p_{1},\ldots ,p_{n}$, the *n* charging stations cover all the sites in *S*. We construct the minimum spanning tree for the vertices with $p_{0}$ as the root. Assume that all the edges in the tree are not longer than 2R. With the initial positions, we can compute the subset of sites covered by each vertex. The main procedure of our method repeatedly relocates the vertices in sequence. When we relocate a vertex, the relocation of all its child vertices needs to be performed beforehand. In the case where two sibling vertices are to be relocated, either of them can be relocated first. With this rule, we start the relocation from the leaf vertices and then up to their parents. Specifically, for a leaf vertex, we find the position for this vertex as discussed in Section 4.2. For a non-leaf vertex, we find the position for the vertex by minimising (21) so that the topology of the constructed tree does not change when the vertex is moved, and the vertex does not lose any sites. After the relocation, the values of *N* and *G* of this vertex are updated by (22) and (23). After the relocation of all the nodes, we update the subset of sites covered by each vertex, as well as the sets *A* and *B*. These procedures repeat until the vertices can no longer be moved. The termination condition is that all the vertices stay at the previous positions in one round of relocation. This method is summarised in Algorithm 1.
**Algorithm 1** Relocating the vertices in the minimum spanning tree     **Input:** $p_{0},p_{1},\ldots ,p_{n}$     **Output:** $p_{1},\ldots ,p_{n}$1:Construct the minimum spanning tree with $p_{0},p_{1},\ldots ,p_{n}$ by taking $p_{0}$ as the root.2:Compute the subset of sites covered by each vertex.3:Construct a relocating sequence.4:**while** Termination condition is unsatisfied **do**5:    **for** Each vertex *j* in the sequence **do**6:        **if** Node *j* is a leaf vertex **then**7:           Find the new position by solving the problem (13) subject to (14) and (15).8:           $N_{j}\leftarrow 0$, $G_{j}\leftarrow 0$.9:        **else**10:           Find the new position by minimising (21) subject to that the topology of the tree does not change when vertex *j* relocates, and vertex *j* does not lose any sites.11:           Update $N_{j}$ and $G_{j}$ by (22) and (23).12:        **end if**13:    **end for**14:    Update the subset of sites covered by each vertex.15:**end while**

For a given set of sites, Algorithm 1 ensures that any movement of a vertex leads to a lower average travel time; we prove this as follows. Algorithm 1 consists of two main procedures. One is the relocation of vertices in sequence, and the other is the update of customers covered by each vertex. For the former, we only relocate one vertex at any time, and we relocate it only if the average travel time reduces for downstream sites impacted by the vertex. As analysed above, such a relocation does not influence upstream sites. Thus, the relocation procedure ensures the decrease in the average travel time. Regarding the latter, for the given locations of any pair of parent-child vertices, the proposed grouping model ensures that any site can be served, and that the travel time for any customer is the lowest. Therefore, Algorithm 1 relocates the vertices to positions with a lower average travel time.

## 5. Simulation Results

In this section, we demonstrate the effectiveness of the proposed algorithms via several typical case studies.

**Figure 5:**
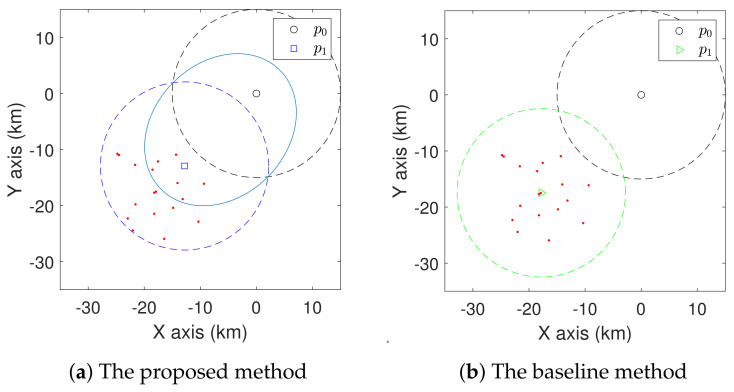
Comparison of the proposed method and baseline method in the first simple case with one charging station. The sites are represented by red dots.

**Figure 6:**
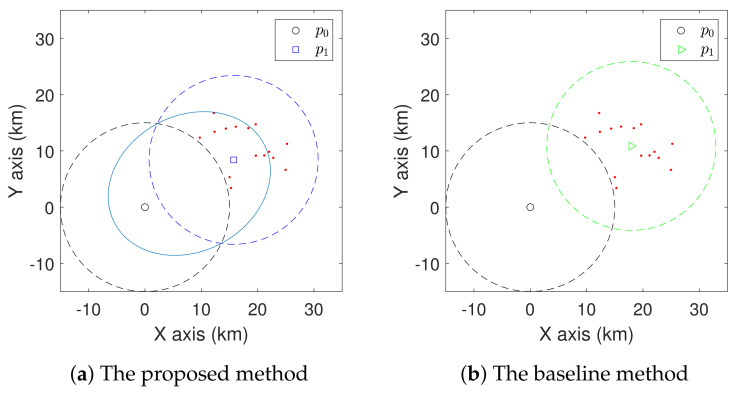
Comparison of the proposed method and baseline method in the second simple case with one charging station. The sites are represented by red dots.

We first show the superiority of the coverage models proposed in Definitions 1 and 2. To better understand the performance, we compare with a baseline method, which groups the sites based on the shortest distance between sites and charging stations, and then moves the charging station to the mass centre of the sites [41]. We consider two simple cases with one charging station to be deployed with some randomly placed sites in Figure 5 and Figure 6. Here, R=15 km. The resulting positions for the charging station found via the proposed method and the baseline method are shown. In the case shown in Figure 5, the average travel distance of the UAV from $p_{0}$ to the sites is 26.2 km for the proposed method. Given a certain flight speed, we can acquire the corresponding travel time. For the same set of sites, the average travel distance achieved by the baseline method is 30.8 km. In the case shown in Figure 6, the proposed method achieves an average travel distance of 21.7 km, while for the same set of sites, the baseline method achieves an average travel distance of 26.6 km. For these cases, we can see that the proposed coverage model leads to a shorter travel distance than the baseline method.

**Figure 7:**
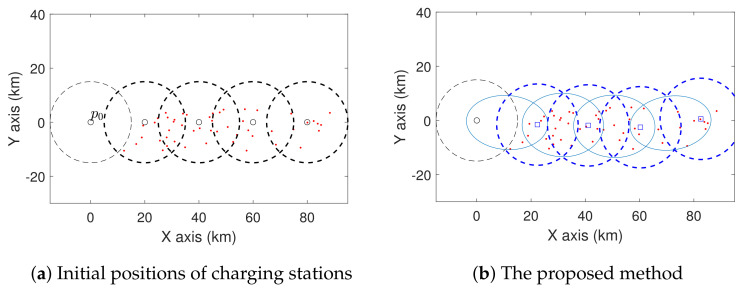

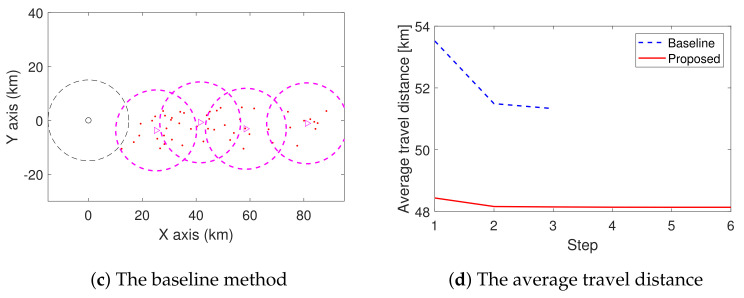
Comparison of the proposed method and baseline method in the case with four charging stations. The sites are represented by red dots.

**Figure 8:**
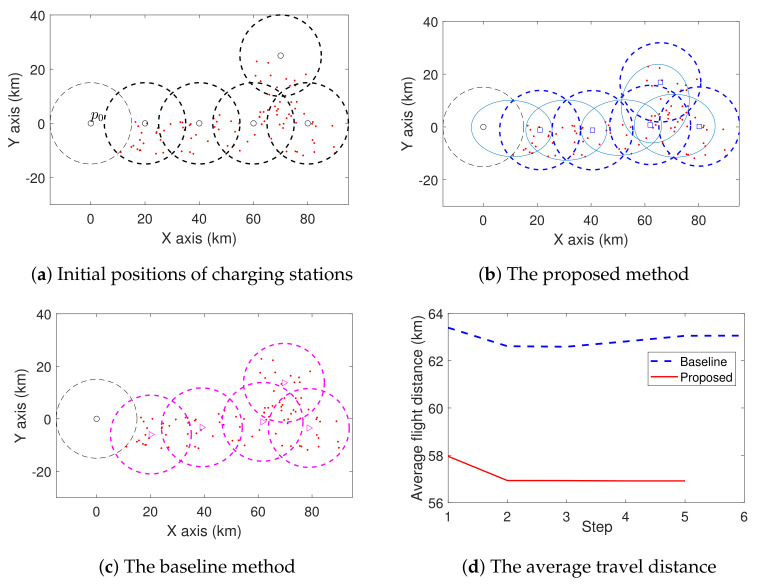
Comparison of the proposed method and baseline method in the case with five charging stations. The sites are represented by red dots.

We also consider some complex cases with more charging stations, and the results are shown in Figure 7 and Figure 8. In Figure 7, we deploy four charging stations using both the proposed method and the baseline method. From the initial positions shown in Figure 7a, these two methods relocate the charging stations to those shown in Figure 7b,c, respectively. Figure 7d shows the average travel distance of UAVs from $p_{0}$ to the sites. Clearly, the proposed method achieves an average lower travel distance. We can also see a similar result in Figure 8.

## 6. Conclusions

In this paper, we considered an approach that exploits PTVs and charging stations to improve UAV flight time. In this approach, a UAV first travels with some PTVs, and then flies through any required charging stations to reach the remote sites to be surveyed. The travel time with PTVs can be estimated using the well-known Monte Carlo method. We mainly investigated the deployment problem of charging stations, which assist UAVs to conduct aerial surveillance. We proposed a new coverage model, which is based on elliptical regions. This model can accurately characterise the travel time of UAVs through the deployed charging stations. Based on such a model, we formulated the deployment problem and proposed a sub-optimal method. This is an iterative method, and we proved that, in each round of relocation, the average travel time of UAVs reduces. The effectiveness of the proposed method has been verified via computer simulations. One limitation of the current approach is that we relocate a charging station by numerically evaluating the candidate sites. An interesting and useful future research direction is to study the analytical solution to this issue.

## Data Availability

Not applicable.

## Abbreviations

The following abbreviations are used in this manuscript:

UAV	Unmanned aerial vehicle
PTV	Public transportation vehicle
QoS	Quality of surveillance
LoS	Line of sight
ILP	Integer linear problem

## Appendix A

**Table A1:** Main symbols used in the paper.

Symbol	Meaning
*D*	Depot of UAVs
*R*	Flight distance corresponding to half of the onboard battery
*n*	Number of charging stations to be deployed
$p_{i}$	Location of charging station *i*
*S*	The set of sites to be surveyed
*s*	Position of site *s*
*G*	Graph formed by vehicle stops, depot, sites and charging stations
$\rho_{s}\left(t\right)$	The probability that the site *s* needs to be surveyed at time *t* of a day
$\lambda_{s}$	The weight of site *s*
τ(u,v,t)	Travel time from node *u* to node *v* when the UAV starts at time *t*
$T_{s}\left(p_{1},\ldots ,p_{n}\right)$	The average travel time of UAV to survey site *s*
